# Risk factors for anaemia among Ghanaian women and children vary by population group and climate zone

**DOI:** 10.1111/mcn.13076

**Published:** 2020-09-18

**Authors:** Nicolai Petry, James P. Wirth, Seth Adu‐Afarwuah, Rita Wegmuller, Bradley A. Woodruff, Sherry A. Tanumihardjo, Helena Bentil, William E.S. Donkor, Thomas N. Williams, Setareh Shahab‐Ferdows, Lilian Selenje, Abraham Mahama, Matilda Steiner‐Asiedu, Fabian Rohner

**Affiliations:** ^1^ GroundWork Fläsch Switzerland; ^2^ Department of Nutrition and Food Science University of Ghana Legon Ghana; ^3^ Department of Nutritional Sciences University of Wisconsin–Madison Madison Wisconsin USA; ^4^ KEMRI‐Wellcome Trust Research Programme Kilifi Kenya; ^5^ USDA Agricultural Research Service Western Human Nutrition Research Center Davis California USA; ^6^ UNICEF Accra Ghana

**Keywords:** anaemia, haemoglobinopathies, inflammation, iron deficiency, malaria, overweight and obesity, vitamin A deficiency

## Abstract

Anaemia has serious effects on human health and has multifactorial aetiologies. This study aimed to determine putative risk factors for anaemia in children 6–59 months and 15‐ to 49‐year‐old non‐pregnant women living in Ghana. Data from a nationally representative cross‐sectional survey were analysed for associations between anaemia and various anaemia risk factors. National and stratum‐specific multivariable regressions were constructed separately for children and women to calculate the adjusted prevalence ratio (aPR) for anaemia of variables found to be statistically significantly associated with anaemia in bivariate analysis. Nationally, the aPR for anaemia was greater in children with iron deficiency (ID; aPR 2.20; 95% confidence interval [CI]: 1.88, 2.59), malaria parasitaemia (aPR 1.96; 95% CI: 1.65, 2.32), inflammation (aPR 1.26; 95% CI: 1.08, 1.46), vitamin A deficiency (VAD; aPR 1.38; 95% CI: 1.19, 1.60) and stunting (aPR 1.26; 95% CI: 1.09, 1.46). In women, ID (aPR 4.33; 95% CI: 3.42, 5.49), VAD (aPR 1.61; 95% CI: 1.24, 2.09) and inflammation (aPR 1.59; 95% CI: 1.20, 2.11) were associated with anaemia, whereas overweight and obese women had lower prevalence of anaemia (aPR 0.74; 95% CI: 0.56, 0.97). ID was associated with child anaemia in the Northern and Middle belts, but not in the Southern Belt; conversely, inflammation was associated with anaemia in both children and women in the Southern and Middle belts, but not in the Northern Belt. Anaemia control programmes should be region specific and aim at the prevention of ID, malaria and other drivers of inflammation as they are the main predictors of anaemia in Ghanaian children and women.

**Key messages**

- Data from a nationally representative cross‐sectional survey were analysed for associations between anaemia and various factors in Ghanaian children and women.
- In Ghana, anaemia was mainly associated with iron and vitamin A deficiency, malaria parasitaemia and inflammation.
- However, factors associated with anaemia are manifold and population group specific and influenced by climatic zones.
- Our results indicate that a multifaceted region‐specific approach is warranted to combat anaemia.

## 1 INTRODUCTION

Anaemia, the state where blood is deficient in haemoglobin or red blood cells, adversely affects human health. Mild forms can manifest as fatigue, weakness and low work productivity (World Health Organization [WHO], 2011c). More severe forms increase the risk for maternal and neonatal mortality, premature birth and delayed child development (WHO, 2017). Despite the implementation of interventions targeted to reduce anaemia over the past decades, anaemia still constitutes about 9% to the total global disability burden (Food and Agriculture Organization of the United Nations [FAO], International Fund for Agricultural Development [IFAD], UNICEF, World Food Programme [WFP], & WHO, 2017), affecting 30–40% of non‐pregnant women of reproductive age, pregnant women and children. From 1995 to 2011, the global prevalence declined only slightly in these population groups in the most seriously affected regions of the world and increased in some others (Stevens et al., 2013). One reason for the rather limited success in tackling anaemia could be its multifactorial nature, the major causes often varying by region or even by country within the same region (Kassebaum et al., 2014). Recent systematic reviews found that, depending on the region, malaria, iron deficiency (ID), genetic blood disorders and infection are strong predictors of anaemia (Engle‐Stone et al., 2017; Petry et al., 2016; Wirth et al., 2017). Hence, it is essential to understand the aetiology of anaemia in a country—and ideally across regions within a country—to successfully implement strategies for its reduction. In Ghana, the prevalence of anaemia was assessed nationally in 2014 as part of a Demographic and Health Survey (Ghana Statistical Service, Ghana Health Service, & ICF International, 2015). More than 65% of children and 42% of women were classified as anaemic, indicating that anaemia posed a severe public health problem according to WHO classification. In this survey, no risk factors of anaemia were determined in children and women, and thus, the targeted formulation of new strategies or the right course for correction of existing strategies to reduce anaemia was difficult.

Thus, the present study was expected to increase the understanding of the predictors of anaemia in Ghana and to allow adjustment of existing national anaemia prevention programmes, as well as to serve as a baseline against which to measure the future progress of such programmes.

## 2 METHODS

### 2.1 Survey design and participants

The Ghana Micronutrient Survey 2017 (GMS 2017) was a cross‐sectional survey with data collection between April and June 2017. To account for Ghana's administrative, agroecological and population density zoning, the following zones were considered as separate *explicit* strata: (1) Southern Belt, consisting predominantly of coastal savannah and rainforest and comprising the Greater Accra, Central, Volta and Western regions; (2) Middle Belt, consisting predominantly of deciduous forest and comprising the Brong‐Ahafo, Ashanti and Eastern regions; and (3) Northern Belt, consisting predominantly of Guinean/Sudanian savannah and comprising the Northern, Upper West and Upper East regions. Sample size calculation is described elsewhere (Wegmüller et al., 2020). A two‐stage sampling procedure with selection of census enumeration areas (EAs) in the first stage with probability of selection being proportional to size was used. In total, 90 EAs were selected. Each of the three *explicit* strata was treated as an independent sample, such that 30 EAs were randomly selected from each stratum. Before the EA selection, 20 implicit strata were created by separating each of Ghana's 10 regions at the time of the survey into urban and rural areas. To ensure that the number of EAs in each implicit stratum approximated the proportion of the population residing in each stratum, the EA list was sorted by implicit stratum prior to calculation of the sampling interval and systematic selection of the EAs. This was done to ensure geographical distribution of the selected EAs and to increase the precision of region‐specific estimates. The survey produced stratum‐representative estimates for preschool children and non‐pregnant women. To account for varying household sizes in the different parts of Ghana (Ghana Statistical Service, 2013), the number of households randomly selected from each EA was different: 29 for the southern and middle strata, 20 for the Upper West and Upper East regions of the northern stratum and 15 for the Northern Region of the northern stratum where average household size was the highest. All households were selected using a random number table. Prior to the household selection, the survey teams conducted a census of all households in that EA to obtain an up‐to‐date household list. Survey teams attempted to recruit all children 6–59 months from each selected household and non‐pregnant women of reproductive age (15–49 years) only in every second selected household.

### 2.2 Data collection and laboratory analysis

Data collection was conducted by experienced field workers who received training on all survey procedures. The training included classroom instruction, hands‐on practice and 2 days of testing in the field.

In selected households, field workers administered the household questionnaire (with modules related to household demographics, sociodemographic variables, and water, sanitation and hygiene [WASH]) to household heads (or any knowledgeable adult household members, if heads were unavailable). The child questionnaire was used to collect information on illnesses in the 2 weeks prior to the interview, dietary diversity and recent consumption of iron or vitamin A supplements and fortified foods in the prior 6 months. The woman questionnaire was used to collect information on marital and occupational status, dietary diversity and iron, vitamin A or folic acid supplement consumption in the prior 6 months.

Survey teams attempted to collect anthropometric measurements and blood samples from all children 6–59 months of age and non‐pregnant women enrolled in the GMS 2017.

#### 2.2.1 Anthropometric measurements

Weight measurement used electronic scales (SECA 877, Hamburg, Germany), with the women and cooperative children standing directly on the scale. Children who could not stand by themselves were measured using the scale's tare function before handing the child to the mother who was already standing on the scale. Children were weighed with only light clothing. Children's lengths and women's heights were measured using the wooden height/length board (UNICEF #S0114540, Copenhagen, Denmark). Children younger than 2 years were measured while in a recumbent position, and older children were measured while standing.

#### 2.2.2 Blood sampling and analyses

For the majority of children, capillary blood was collected. Venous blood was collected in a subsample of children 18–59 months of age (about 15% of total children) who were selected for additional blood analyses (not presented here), which required a larger quantity of blood. Capillary blood was collected by either finger prick (children 12 months of age and older) or heel prick (children 6–11 months of age). Following puncture with a lancet (Becton Dickinson, Franklin Lakes, NJ, USA), the first two drops were wiped away, and the third and fourth drops of blood were collected to measure haemoglobin concentration and malaria status, respectively. Thereafter, approximately 300–400 μl of blood was collected into a silica‐coated Microtainer™ for later analysis. Venipuncture was done in all consenting non‐pregnant women. For venipuncture in women and children, blood was collected into 6‐ml PET serum tubes containing clotting activator (Becton Dickinson, Franklin Lakes, NJ, USA). Using a DIFF‐SAFE device (Becton Dickinson, Franklin Lakes, NJ, USA), a small amount of blood was immediately extracted from the tubes onto a weighing boat to determine haemoglobin concentration and malaria status. Directly after blood collection, microtainers and blood collection tubes were placed into a dark cold box at ~4°C. All whole blood samples were centrifuged at 3,000 rpm for 7 min on the same day as blood drawing, and serum was separated and aliquoted for later analyses. Serum aliquots and pellets were stored at −20°C for the duration of field work. At the end of survey data collection, samples were consolidated and stored at the University of Ghana at −20°C until shipment on dry ice to international laboratories.

Haemoglobin concentration was measured using a portable haemoglobinometer (Hb301+, HemoCue, Ängelholm, Sweden). Quality control of the HemoCue™ devices was conducted and recorded on a daily basis using control materials (Eurotrol 301 low and normal level, Ede, the Netherlands). For on‐site malaria measurements, a qualitative immunochromatographic rapid diagnostic test was used to detect *Plasmodium falciparum* specifically and *Plasmodium* species in general including *Plasmodium vivax*, *Plasmodium malariae* or *Plasmodium ovale* (Standard Diagnostics Inc, Gyeonggi‐do, Republic of Korea).

Serum for all children 6–59 months of age and all women was analysed in one run for retinol‐binding protein (RBP), serum ferritin (SF), soluble transferrin receptor (sTfR), C‐reactive protein (CRP) and α1‐acid glycoprotein (AGP) at the VitMin Lab (Willstätt, Germany) using a sandwich enzyme‐linked immunosorbent assay (ELISA) method (Erhardt, Estes, Pfeiffer, Biesalski, & Craft, 2004).

Serum folate and vitamin B_12_ concentrations were measured quantitatively in one half of the samples obtained from non‐pregnant women by using a Cobas e411 analyzer (Roche Diagnostics, Indianapolis, IN, USA) at the USDA/ARS Western Human Nutrition Research Center (Davis, USA).

Genetic blood disorder (sickle cell disease [HbSS] and trait [HbAS] and α‐thalassemia) analyses were done in all children 6–59 months of age and half of non‐pregnant women at the KEMRI‐Wellcome Trust Research Programme (Kilifi, Kenya) using polymerase chain reaction as described elsewhere (Atkinson et al., 2006; Chong, Boehm, Higgs, & Cutting, 2000; Waterfall & Cobb, 2001).

### 2.3 Indicators and clinical thresholds

Haemoglobin concentrations were adjusted for elevation (for women and children) and smoking status (for women only) as recommended by WHO (2011d). Children with haemoglobin concentrations <110 g/L were classified as anaemic, and concentrations <70, 70–99 and 100–109 g/L denoted severe, moderate and mild anaemia, respectively. For non‐pregnant women, a haemoglobin concentration of <120 g/L was used to define anaemia, and concentrations of <80, 80–109 and 110–119 g/L denoted severe, moderate and mild anaemia, respectively (WHO, 2011d). Using weighted anaemia prevalence in children and women, WHO (2011d) criteria were used to assess the public health significance (mild, moderate or severe) implicated by anaemia in children and women.

Cut‐offs defining elevated CRP and AGP were >5 mg/L and >1 g/L, respectively. Subclinical inflammation and no inflammation was categorized in four groups: no inflammation, elevated CRP only, elevated CRP and AGP, and elevated AGP only (Thurnham et al., 2008). Using CRP and AGP, SF (Thurnham et al., 2010) and RBP (Thurnham, McCabe, Northrop‐Clewes, & Nestel, 2003) were adjusted for inflammation according to Thurnham. SF concentrations <12 and <15 μg/L were used to define ID in children and women, respectively (WHO, 2011a). In children, vitamin A deficiency (VAD) was defined as RBP < 0.7 μmol/L, and RBP < 1.05 μmol/L was used to define vitamin A insufficiency in women (WHO, 2009). As there were few individuals with sickle cell disease, sickle cell disease and sickle cell trait were grouped together into a single category to examine the association between these genetic blood disorder and anaemia.

Vitamin B_12_ concentrations were classified as ‘deficient’ when serum concentration was <148 pmol/L and ‘marginal’ when between 148 and 220 pmol/L (Brito, Mujica‐Coopman, López de Romaña, Cori, & Allen, 2015; Institute of Medicine, 2000). As recommended by WHO (Allen, DeBenoist, Dary, & Hurrell, 2006), folate deficiency was defined as serum folate concentrations <10 nmol/L.

Undernutrition (including wasting, stunting and underweight) and overnutrition were calculated for all children using the WHO Growth Standard (WHO Multicentre Growth Reference Study Group, 2006), and *z* scores below −2.0 were used to classify a child as stunted, wasted or underweight. Overweight in children was defined as a weight‐for‐height *z* score greater than +2.0 but less than or equal to +3.0, and obesity was defined as a weight‐for‐height *z* score greater than +3.0.

Chronic energy deficiency and overnutrition in non‐pregnant women were assessed using body mass index (BMI). Cut‐off points for BMI were as follows: <16.0 severe chronic energy deficiency; 16.0–16.9 moderate chronic energy deficiency; 17.0–18.4 at risk for energy deficiency; 18.5–24.9 normal; 25.0–29.9 overweight; and ≥30.0 obese (Shetty & James, 1994).

### 2.4 Data management and statistical analysis

Direct electronic data entry was done by using Open Data Kit (ODK) loaded on tablet computers. Data analysis was done by using Stata/IC Version 14.2. Statistical weights were applied to correct for the unequal probability of selection in the three strata.

Individual characteristics and prevalence of nutritional and micronutrient deficiencies were calculated in aggregate (i.e., for the entire sample across all strata), for each stratum separately and for sex (for children) and age subgroups in women and children. The statistical precision of all prevalence estimates was assessed using 95% confidence intervals (CIs), which were calculated accounting for the complex sampling, including the cluster and implicit and explicit stratified sampling used in this survey.

For each target group, only variables measured in all subjects were considered for inclusion into multivariate models. Bivariate associations between factors and anaemia prevalence were examined separately at the national level and for each stratum (Tables S1–S3). Factors associated with anaemia in bivariate analyses with a *P* value <0.1 were considered eligible for inclusion into the multivariate models, as ‘more traditional levels such as 0.05 can fail in identifying variables known to be important’ (Bursac, Gauss, Williams, & Hosmer, 2008). The variables in the initial models were examined for colinearity by calculating the variance inflation factor (VIF) for all independent variables; a VIF ≥ 4 was used as a threshold for colinearity (Hair, Black, Babin, & Anderson, 2014). Following the construction of the initial models, backward elimination was used to remove variables that were not statistically significantly (*P* < 0.05) associated with anaemia. The regression models produced adjusted prevalence ratios (aPRs). The aPRs produced during the final regression model and the proportion of anaemia cases with exposure to the risk factor (pd) were used to calculate the population attributable fraction for each covariate using the following calculation (Rockhill, Newman, & Weinberg, 1998):

$$\mathrm{pd}=\frac{\left(\mathrm{aRR}‐1\right)}{\left(\mathrm{arr}\right)}.$$

### 2.5 Ethical considerations

Ethical approval for the survey was obtained from Ghana Health Service Ethics Review Committee (GHS‐ERC Number: GHS‐ERC: 15/01/2017). The survey protocol was also registered with the Open Science Framework study registry (DOI: 10.17605/OSF.IO/J7BP9). Oral consent was sought from the respondent of the household interview. Selected women and caregivers of participating children were asked to provide written informed consent for interview, anthropometric measurements and phlebotomy. If potential survey participants were illiterate, the consent form was read out loud to them and a thumbprint or fingerprint plus a witness signature was taken as evidence of consent in lieu of a signature. Survey respondents diagnosed with severe anaemia, malaria or severe acute malnutrition were referred for further diagnosis and treatment at the local health facility.

## 3 RESULTS

The survey sample included 2,123 households, 1,053 women and 1,234 children. A blood sample was provided by 973 women and 1,165 children. Basic demographic characteristics of the households, women and children have been presented elsewhere (University of Ghana, GroundWork, University of Wisconsin–Madison, KEMRI‐Wellcome Trust, & UNICEF, 2017).

All variables with *P* values <0.1 in bivariate analyses were included in the initial multivariate models for children and women (Tables S1–S3). In the initial child models, no colinearity was observed, and all independent covariates had a VIF < 2.5. Mean VIF in the national, southern, middle and northern child models were 1.39, 1.47, 1.28 and 1.15, respectively. There was also no colinearity found in the initial women models, and all independent variables had a VIF < 1.5. Mean VIF in the national, southern, middle and northern women models were 1.18, 1.03, 1.02 and 1.06, respectively.

Following backward elimination, only factors with a *P* value <0.05 were retained in each parsimonious model. In national models for children and women, ‘stratum’ remained as a covariate following the backward elimination procedure, but was manually removed as geographical location can only influence disease state by acting through other factors, and is thus not directly linked to anaemia.

As shown in Table 1 for the national model, malaria, ID and inflammation together account for almost one half of cases of anaemia in children. Further, in children, ID had the highest aPR of all factors investigated. Moreover, children who had fever in the 2 weeks preceding the survey, who were stunted or who were vitamin A deficient were more likely to have anaemia.

**TABLE 1:** Adjusted prevalence ratio of anaemia and population attributable fraction in children 6–59 months, Ghana 2017

Characteristic	Category	Adjusted prevalence ratio	95% CI	Population attributable fraction^a^
National model (*n* = 1,084)^b^				
Iron status^c^	Deficient	2.20	(1.88, 2.59)	19.1%
	Not deficient	Referent	—	
Vitamin A status^d^	Deficient	1.38	(1.19, 1.61)	8.2%
	Not deficient	Referent	—	
Malaria parasitaemia^e^	Positive	1.96	(1.65, 2.32)	15.1%
	Negative	Referent	—	
Inflammation^f^	Yes	1.26	(1.08, 1.46)	12.3%
	No	Referent	—	
Fever in the past 2 weeks	Yes	1.27	(1.1, 1.48)	9.1%
	No	Referent	—	
Stunting	Stunted	1.26	(1.09, 1.46)	5.5%
	Not stunted	Referent	—	
Southern stratum model (*n* = 318)^b^				
Malaria parasitaemia^e^	Positive	1.92	(1.38, 2.68)	21.9%
	Negative	Referent	—	
Inflammation^f^	Yes	1.86	(1.24, 2.79)	33.5%
	No	Referent	—	
Fever in the past 2 weeks	Yes	1.49	(1.05, 2.13)	19.3%
	No	Referent	—	
Middle stratum model (*n* = 383)^b^				
Iron status^c^	Deficient	2.75	(1.85, 4.11)	18.3%
	Not deficient	Referent	—	
Inflammation	Yes	2.09	(1.47, 2.97)	36.7%
	No	Referent	—	
Malaria parasitaemia^e^	Positive	2.36	(1.60, 3.49)	21.2%
	Negative	Referent	—	
Residence	Rural	1.54	(1.06, 2.23)	23.3%
	Urban	Referent	—	
Northern stratum model (*n* = 398)^b^				
Iron status^c^	Deficient	1.75	(1.45, 2.11)	23.3%
	Not deficient	Referent	—	
Stunting	Stunted	1.24	(1.04, 1.48)	6.2%
	Not stunted	Referent	—	
Malaria parasitaemia^e^	Positive	1.76	(1.39, 2.23)	6.3%
	Negative	Referent	—	
Cough in the past 2 weeks	Yes	1.21	(1.02, 1.42)	5.2%
	No	Referent	—	

Abbreviation: CI, confidence interval.

^a^ Calculated using relative risk from Poisson regression.

^b^ Child's age in months as a continuous covariate was included in all regression models.

^c^ Iron deficiency defined as serum ferritin <12 μg/L.

^d^ Vitamin A deficiency defined as retinol‐binding protein <0.7 μmol/L.

^e^ Malaria status identified using rapid diagnostic tests during Ghana Micronutrient Survey data collection.

^f^ Inflammation defined as elevated C‐reactive protein (>5 mg/L) and/or elevated α1‐acid glycoprotein (>1 g/L).

The investigation of putative risk factors for anaemia by stratum showed that for the middle stratum, almost all anaemia was associated with the assessed factors (>99%), whereas for the southern and northern strata, this was the case only for 75% and 41% of total anaemia, respectively. In all three strata, malaria parasitaemia has one of the strongest associations with anaemia. Iron status and inflammation each was included in the model in two out of the three strata. No association between iron status and anaemia was found in the southern stratum, and inflammation was not associated with anaemia in the northern stratum. Other risk factors, such as fever, cough, rural or urban residency, and stunting were retained in only one stratum‐specific model and had weaker associations with anaemia.

For women, the predominant risk factor for anaemia was ID, explaining about one third of the total anaemia prevalence nationally (Table 2). About 10% of the total anaemia in women was ascribed to each of vitamin A status and inflammation. On the other hand, women with obesity or overweight had a lower risk of anaemia.

**TABLE 2:** Adjusted prevalence ratio of anaemia and population attributable fraction in non‐pregnant women 15–49 years, Ghana 2017

Characteristic	Category	Adjusted prevalence ratio	95% CI	Population attributable fraction^a^
National model (*n* = 969)				
Inflammation^b^	Yes	1.59	(1.2, 2.1)	10.1%
	No	Referent	—	
Vitamin A status^c^	Insufficient	1.61	(1.24, 2.09)	9.9%
	Sufficient	Referent	—	
Iron status^d^	Deficient	4.33	(3.42, 5.49)	32.8%
	Not deficient	Referent	—	
Overweight or obesity	Yes	0.74	(0.56, 0.97)	−11.0%
	No	Referent	—	
Southern stratum model (*n* = 297)				
Inflammation^b^	Yes	1.63	(1.05, 2.53)	11.2%
	No	Referent	—	
Iron status^d^	Deficient	4.00	(2.80, 5.73)	28.9%
	Not deficient	Referent	—	
Overweight or obesity	Yes	0.54	(0.35, 0.84)	−29.1%
	No	Referent	—	
Middle stratum model (*n* = 397)				
Inflammation^b^	Yes	1.86	(1.18, 2.93)	18.0%
	No	Referent	—	
Vitamin A status^c^	Insufficient	2.09	(1.22, 3.6)	11.4%
	Sufficient	Referent	—	
Iron status^d^	Deficient	4.54	(2.84, 7.26)	28.9%
	Not deficient	Referent	—	
Northern stratum model (*n* = 398)				
Vitamin A status^c^	Insufficient	1.77	(1.17, 2.67)	19.9%
	Sufficient	Referent	—	
Iron status^d^	Deficient	4.02	(2.64, 6.13)	42.7%
	Not deficient	Referent	—	

Abbreviation: CI, confidence interval.

^a^ Calculated using relative risk from Poisson regression.

^b^ Inflammation defined as elevated C‐reactive protein (>5 mg/L) and/or elevated α1‐acid glycoprotein (>1 g/L).

^c^ Vitamin A insufficiency defined as retinol‐binding protein <1.05 μmol/L.

^d^ Iron deficiency defined as serum ferritin <15 μg/L.

Similar to children, putative risk factors for women remaining in the final stratum‐specific models were different among the strata. ID was associated with anaemia in the three strata and produced the largest proportion of anaemia in all strata. Inflammation and VAD were important risk factors in two of the three strata where they accounted for between 10% and 20% of all anaemia and women. Obesity or overweight reduced the likelihood of anaemia by about 50% for women living in the southern stratum, but not for women living in the other parts of Ghana.

## 4 DISCUSSION

The data presented here suggest that multiple factors contribute to anaemia in Ghanaian children and women and that these factors differ by stratum, which may reflect differences in agroecological conditions and consequently indicate varying dietary habits and disease patterns.

Nationally, anaemia in children is mainly associated with ID, malaria, inflammation and VAD, whereas in women, anaemia is predominantly associated with ID, vitamin A insufficiency and inflammation. ID is the single major contributor to anaemia, with almost one fifth of anaemia in children and one third of anaemia in women associated with it. Nonetheless, ID is associated with a smaller fraction of anaemia than the 50% often assumed (WHO, 2017). It is, however, close to a more recent estimate of 25% for children and 37% for women (Petry et al., 2016).

About 10% of total anaemia can be ascribed to VAD in children and to vitamin A insufficiency in women. Vitamin A insufficiency (<1.05 μmol/L) was used in the multivariate model for women instead of the WHO‐recommended cut‐off for VAD of 0.7 μmol/L (WHO, 2011b) because it might be a more suitable definition of adequate vitamin A status in women (Tanumihardjo, 2010). Several mechanisms exist by which VAD reduces haemoglobin concentration, including increased frequency and severity of infectious diseases and poor mobilization of iron stores from tissues (Semba & Bloem, 2002; Zimmermann et al., 2006). Our analysis shows that a direct effect, likely independent of these mechanisms, is substantial in Ghanaian children and women. Therefore, public health decision makers should include vitamin A provision in the range of activities to prevent and treat anaemia.

Unlike vitamin A insufficiency, neither folate nor vitamin B_12_ deficiencies, the leading causes of megaloblastic anaemias (Green & Datta Mitra, 2017), are positively associated with anaemia in Ghanaian women. Notwithstanding, overall, other micronutrient deficiencies investigated in this survey (i.e., VAD and ID) account for more than 40% of the anaemia in women, compared with only one quarter in children.

Stratum or place of residence cannot have a direct influence on anaemia; it must operate through other factors, such as prevalence and type of disease vectors, dietary patterns, agricultural practices and efficiency, climate and others. The separate stratum‐specific models clearly show different constellations of putative risk factors within each stratum.

The prevalence of anaemia associated with nutritional factors is especially high in the northern stratum. The northern part of Ghana consists mainly of savannah, with a predominantly dry climate, and thus, it is possible that a larger proportion of the population being affected by inadequate nutrition compared with other parts of the country, whereas malaria transmission may be less intense. This is also reflected in the high prevalence of wasting, stunting and underweight in children and the low prevalence of overweight and obesity in women as well as the higher prevalence of ID and VAD in children and women in the northern stratum compared with the other strata (University of Ghana et al., 2017). In Ghana, the majority of the population subsists on a plant‐based diet, which is low in haem iron (Lombardi‐Boccia, Martinez‐Dominguez, & Aguzzi, 2002) and high in iron absorption inhibitors and thus facilitates the development of ID anaemia (McLean, Cogswell, Egli, Wojdyla, & De Benoist, 2009). Insufficient dietary intake of iron, as well as other micronutrients, could be addressed by fortification of staple foods; however, data from our survey indicate that existing fortification programmes perform poorly. Fewer than 10% of households had adequately fortified wheat flour, and about one half of households had adequately fortified vegetable oil at the time of the survey (University of Ghana et al., 2017). Strengthening these already existing fortification programmes could increase dietary intake of iron and vitamin A and thus reduce anaemia.

Malaria and inflammation were found to be associated with anaemia in Ghanaian children, an association that has also been reported from other countries with high burden of infection and high malaria prevalence (Engle‐Stone et al., 2017; Ministry of Health and Sanitation (Sierra Leone), Helen Keller International, UNICEF, & WHO, 2015). In women, inflammation contributes to about 10% of total anaemia, whereas malaria is not associated with anaemia at all. This difference might partly be due to acquired immunity in the women, which may blunt the inflammatory response to malaria infection as well as the infection itself (Cercamondi et al., 2010). However, in Ghanaian children with malaria or inflammation, anaemia prevalence is almost twice as high, compared with those without malaria or inflammation. Multivariate analyses show that nationally almost one third of total child anaemia can be attributed to either malaria or anaemia of chronic disease, which are not clearly distinguishable as they partly cause anaemia through similar pathways and might have a compounding effect. Chronic diseases and malaria trigger anaemia by the hepatic secretion of the peptide hormone hepcidin (Girelli, Nemeth, & Swinkels, 2016; Wang & Babitt, 2016). In addition, both cause haemolytic anaemia, erythropoietic suppression and dyserythropoiesis (Cowman, Tonkin, Tham, & Duraisingh, 2017; Lamikanra et al., 2007; Nemeth & Ganz, 2014). The association between both malaria and anemia, and inflammation and anaemia is particularly strong in the southern stratum, which has a tropical climate in which the risk of malaria exposure is high. In such areas, programmes targeting at malaria transmission reduction, such as mosquito net distribution, would also help prevent anaemia.

In the developing world, inflammation and infectious diseases are often related to poor sanitary conditions (Cumming & Cairncross, 2016). Constant exposure to poor sanitary conditions, in particular a constant faecal oral contamination, can damage intestinal villi and cause chronic inflammation (McKay, Gaudier, Campbell, Prentice, & Albers, 2010), and consequently weaken the immune system, and contribute to malnutrition and growth faltering (Crane, Jones, & Berkley, 2015). Although in the southern stratum, poor sanitary conditions are significantly associated with anaemia in the bivariate analyses, this association is no longer found in the multivariate model. Nevertheless, improving household sanitary conditions could help to reduce inflammation and subsequently reduce the prevalence of anaemia of chronic disease. As inflammation is significantly associated with anaemia in most of the child and women models, further analyses examining the putative risk factors of inflammation are warranted to determine if—and to which extent—sanitation and hygiene variables are associated with inflammation.

In addition to malaria and inflammation, anaemia in children is associated with fever and cough in the past 2 weeks prior to the interview in the different strata, possibly representing underlying exposure to varying disease vectors. Fever in the past 2 weeks, which is associated with anaemia in the southern stratum, may be related to malaria, as nearly one quarter of children are affected with malaria in that region of Ghana (University of Ghana et al., 2017). Low‐density malaria infections, which are not identified by malaria rapid diagnostic tests, have been associated with fever in other countries (McCreesh et al., 2018). In contrast, cough in the past 2 weeks is associated with anaemia only in the northern stratum. Recent cough may represent a proxy for acute lower respiratory infection, which has been associated with anaemia in other studies (Wirth et al., ). It must be noted however, that cough, diagnosed subjectively via caretakers, is an imprecise indicator and findings related to this objective measure should be interpreted with caution (Shields, Bush, Everard, McKenzie, & Primhak, 2008).

Overweight and obesity have a protective effect against anaemia in women. This is surprising because other studies show that obesity contributes to ID (Cepeda‐Lopez et al., 2011) by causing low‐grade inflammation that blocks iron uptake (Cepeda‐Lopez, Melse‐Boonstra, Zimmermann, & Herter‐Aeberli, 2015). However, it is possible that overweight and obese women have a lower risk of developing nutritionally induced anaemia as they might eat larger amounts and a wider variety of foods, including iron‐rich foods, compared with those who are not overweight or obese. In addition, overweight and obesity in our study are more common in women with high socio‐economic status who may have other characteristics not measured in the survey that are associated with better nutritional status. Thus, this finding may be due to uncorrected confounding by unmeasured variables.

Overall, only 69% and 53% of the anaemia in children and women, respectively, could be explained by the factors included in the analyses. The contribution of other common factors in sub‐Saharan African countries, such as schistosomiasis or hookworm (Kassebaum et al., 2014), should be further explored.

Lastly, it is important to note the inherent challenge in using cross‐sectional data to draw causal inferences by identifying risk factors—and calculating relative risks—of various health conditions, such as anaemia. Although strictly speaking, the GMS did not measure risk, ‘prevalence ratios’ calculated from the ratio of prevalence rates were treated as equivalent to relative risk. The relative risk is most commonly calculated as the ratio of cumulative incidence rates over a specified time period. The putative relative risks and the outcome anaemia considered in this survey are of such long duration that a cumulative incidence approaches the prevalence. As a result, the prevalence ratio is a good approximation of a relative risk. In addition, the putative risk factors included in this analysis have been demonstrated to increase the risk of anaemia in multiple studies, including cohort studies and randomized controlled trials, which provide very strong evidence for causality (Chaparro & Suchdev, 2019; Kassebaum et al., 2016). As a result, there is little question that these factors are true risk factors for anaemia in Ghana. This study does not attempt to prove that these factors are truly risk factors; it measures the relative contribution of these known risk factors for anaemia in the specific population of Ghanaian women and children.

## 5 CONCLUSION

The current analysis illustrates that no single factor accounts for a very large proportion of all cases of anaemia in Ghana, particularly in children, and that the contribution of anaemia risk factors varies by region. Thus, a multifaceted approach is warranted to tackle anaemia in Ghana. In the Northern Belt, anaemia is mainly associated with nutritional factors; programmes should therefore include the promotion of age‐appropriate infant and young child feeding practices, including the promotion of foods (fortified or unfortified) rich in iron and vitamin A. Also, in women, programmes to promote the consumption of iron‐rich foods and iron supplements should be considered. For the middle and southern strata, programmes should focus more on malaria prevention and WASH interventions.

## CONFLICTS OF INTEREST

The authors declare no competing interests. The authors alone are responsible for the views expressed in this publication, and they do not necessarily represent the decisions, policy or views of UNICEF.

## CONTRIBUTIONS

NP, JPW, SA‐A, RW, BAW, LS, AM, MS‐A and FR designed the survey. NP, JPW, SA‐A, RW, BAW, SAT, HB, WESD, MS‐A and FR managed the data collection. SAT, TNW and SS‐F conducted thelaboratory analyses. NP, JPW, BAW and FR analysed the data. NP and FR wrote the first draft of the paper and had primary responsibility for the final content. All authors read and approved the final manuscript.

## Supporting information


**Table S1.** Bivariate associations between various risk factors and anemia in children in Ghana, National results
**Table S2.** Bivariate associations between various risk factors and anemia in children in Ghana, South Stratum results
**Table S3.** Bivariate associations between various risk factors and anemia in children in Ghana, Middle Stratum results
**Table S4.** Bivariate associations between various risk factors and anemia in children in Ghana, North Stratum results
**Table S5.** Bivariate associations between various risk factors and anemia in women in Ghana, National results
**Table S6.** Bivariate associations between various risk factors and anemia in women in Ghana, South Stratum results
**Table S7.** Bivariate associations between various risk factors and anemia in women in Ghana, Middle Stratum results
**Table S8.** Bivariate associations between various risk factors and anemia in women in Ghana, North Stratum results
