# Supplementary material for: Risk factors for anaemia among Ghanaian women and children vary by population group and climate zone
Source: Matern Child Nutr. 2020 Sep 18;17(2):e13076. doi: 10.1111/mcn.13076 (PMC7988882; doi:10.1111/mcn.13076)
Supplement: Supplementary file 1 — Table S1. Bivariate associations between various risk factors and anemia in children in Ghana, National results Table S2. Bivariate associations between various risk factors and anemia in children in Ghana, South Stratum results Table S3. Bivariate associations between various risk factors and anemia in children in Ghana, Middle Stratum results Table S4. Bivariate associations between various risk factors and anemia in children in Ghana, North Stratum results Table S5. Bivariate associations between various risk factors and anemia in women in Ghana, National results Table S6. Bivariate associations between various risk factors and anemia in women in Ghana, South Stratum results Table S7. Bivariate associations between various risk factors and anemia in women in Ghana, Middle Stratum results Table S8. Bivariate associations between various risk factors and anemia in women in Ghana, North Stratum results [file MCN-17-e13076-s001.docx]

**Supplementary material**

Supplementary Table 1. Bivariate associations between various risk factors and anemia in children in Ghana, National results

| ***Characteristic*** | ***Number of children in group*** | ***% Anemia ^a, b^*** | ***(95% CI) ^c^*** | ***P value ^d^*** |
| --- | --- | --- | --- | --- |
| **Age Group (in months)** |  |  |  |  |
| 6-11 | 120 | 44.8 | (36.0; 53.9) | **<0.0001** |
| 12-23 | 276 | 46.1 | (39.1; 53.3) |  |
| 24-35 | 256 | 36.7 | (30.3; 43.7) |  |
| 36-47 | 265 | 30.2 | (22.9; 38.6) |  |
| 48-59 | 253 | 23.4 | (18.1; 29.6) |  |
| **Sex** |  |  |  |  |
| Male | 581 | 38.4 | (33.5; 43.5) | **0.0452** |
| Female | 589 | 32.8 | (28.4; 37.6) |  |
| **Sickle cell status** |  |  |  |  |
| Normal | 990 | 35.6 | (31.7; 39.8) | 0.4814 |
| Sickle cell trait (HbAS) or disease (HbSS) | 142 | 32.6 | (25.2; 41.0) |  |
| **α-thalassemia** |  |  |  |  |
| Normal | 727 | 32.1 | (28.2; 36.3) | **0.0269** |
| α-thalassemia heterozygote/homozygote | 350 | 41.6 | (34.4; 49.3) |  |
| **Vitamin A deficient** ^e^ |  |  |  |  |
| No (RBP≥0.7 μmol/L) | 919 | 31.3 | (27.9; 35.0) | **<0.0001** |
| Yes (RBP<0.7 μmol/L) | 240 | 49.6 | (42.1; 57.1) |  |
| **Iron deficient** ^e^ |  |  |  |  |
| No (ferrtin≥12 μg/L) | 882 | 29.2 | (25.5; 33.1) | **<0.0001** |
| Yes (ferritin<12 μg/L) | 277 | 57.1 | (50.4; 63.5) |  |
| **Stunting** |  |  |  |  |
| No (HAZ ≥-2SD) | 898 | 33.0 | (28.7; 37.5) | **0.0068** |
| Yes (HAZ <-2SD) | 257 | 43.7 | (37.1; 50.6) |  |
| **Wasting** |  |  |  |  |
| No (WHZ ≥-2SD) | 1059 | 35.0 | (31.0; 39.2) | 0.4362 |
| Yes (WHZ <-2SD) | 89 | 39.7 | (28.8; 51.7) |  |
| **Underweight** |  |  |  |  |
| No (WHZ ≥-2SD) | 972 | 34.2 | (30.4; 38.3) | **0.0488** |
| Yes (WHZ <-2SD) | 185 | 42.8 | (34.5; 51.6) |  |
| **Inflammation categories** ^f^ |  |  |  |  |
| None | 640 | 26.2 | (22.2; 30.7) | **<0.0001** |
| Elevated CRP only | 39 | 47.5 | (29.8; 65.8) |  |
| Elevated CRP and AGP | 198 | 52.2 | (45.4; 58.9) |  |
| Elevated AGP only | 282 | 40.9 | (34.7; 47.4) |  |
| **Any inflammation** |  |  |  |  |
| None | 640 | 26.2 | (22.2; 30.7) | **<0.0001** |
| Any inflammation | 519 | 45.6 | (40.9; 50.5) |  |
| **Malaria status** ^g^ |  |  |  |  |
| Negative | 879 | 31.2 | (27.0; 35.6) | **<0.0001** |
| Positive | 233 | 54.7 | (47.4; 61.8) |  |
| **Diarrhea in past 2 weeks** |  |  |  |  |
| No | 882 | 33.8 | (29.7; 38.0) | **0.0371** |
| Yes | 289 | 41.2 | (34.4; 48.2) |  |
| **Cough in past 2 weeks** |  |  |  |  |
| No | 864 | 35.4 | (31.0; 40.2) | 0.7892 |
| Yes | 307 | 36.0 | (29.4; 43.2) |  |
| **Fever in past 2 weeks** |  |  |  |  |
| No | 733 | 30.8 | (26.9; 35.0) | **<0.0001** |
| Yes | 399 | 45.2 | (39.3; 51.2) |  |
| **Lower respiratory infection in past 2 weeks** |  |  |  |  |
| No | 1144 | 35.1 | (31.2; 39.3) | **0.0176** |
| Yes | 28 | 57.9 | (40.1; 73.9) |  |
| **Household sanitation** |  |  |  |  |
| Unimproved | 1036 | 36.5 | (32.8; 40.5) | **0.0770** |
| Improved | 136 | 29.1 | (21.9; 37.9) |  |
| **Household owns soap** |  |  |  |  |
| No | 109 | 39.7 | (31.2; 48.7) | 0.4696 |
| Yes | 886 | 36.0 | (31.7; 40.6) |  |
| **Household has water at handwashing site** |  |  |  |  |
| No | 390 | 36.9 | (31.7; 42.4) | 0.7912 |
| Yes | 605 | 36.0 | (31.0; 41.3) |  |
| **Household wealth quintile** |  |  |  |  |
| Lowest | 420 | 47.0 | (40.3; 53.8) | **<0.0001** |
| Second | 247 | 36.4 | (28.5; 45.2) |  |
| Middle | 213 | 38.8 | (32.4; 45.6) |  |
| Fourth | 154 | 28.3 | (20.7; 37.3) |  |
| Highest | 138 | 13.8 | (9.0; 20.6) |  |
| **Stratum** |  |  |  |  |
| Southern Belt | 323 | 32.3 | (25.2; 40.2) | **<0.0001** |
| Middle Belt | 447 | 28.2 | (22.1; 35.3) |  |
| Northern Belt | 402 | 53.2 | (46.6; 59.8) |  |
| **Residence** |  |  |  |  |
| Urban | 432 | 26.8 | (21.1; 33.4) | **0.0007** |
| Rural | 740 | 42.1 | (37.3; 47.0) |  |
| Note: The n’s are un-weighted denominators for each subgroup; subgroups that do not sum to the total have missing data of missing data. | | | | |
| ^a^ Percentages weighted for unequal probability of selection. | | | | |
| ^b^ Anemia defined as hemoglobin < 110 g/L. | | | | |
| ^c^ CI=confidence interval, calculated taking into account the complex sampling design. | | | | |
| ^d^ Chi-square p-value <0.05 indicates that the proportion in at least one subgroup is statistically significantly different from the values in the other subgroups | | | | |
| ^e^ RBP [18] and ferritin [17] values adjusted for inflammation using Thurnham approach | | | | |
| ^f^ Elevated CRP and AGP defined as > 5mg/ L and >1 g/L, respectively | | | | |
| ^g^ Positive malaria status identified using rapid diagnostic tests during GMS data collection | | | | |

Supplementary Table 2. Bivariate associations between various risk factors and anemia in children in Ghana, South Stratum results

| ***Characteristic*** | ***Number of children in group*** | ***% Anemia ^a, b^*** | ***(95% CI) ^c^*** | ***P value ^d^*** |
| --- | --- | --- | --- | --- |
| **Age Group (in months)** |  |  |  |  |
| 6-11 | 37 | 35.2 | (19.8; 54.4) | **0.0306** |
| 12-23 | 67 | 49.3 | (33.3; 65.5) |  |
| 24-35 | 68 | 30.5 | (21.9; 40.8) |  |
| 36-47 | 74 | 22.4 | (12.9; 36.0) |  |
| 48-59 | 77 | 25.1 | (14.4; 40.0) |  |
| **Sex** |  |  |  |  |
| Male | 165 | 31.7 | (22.8; 42.1) | 0.8002 |
| Female | 158 | 32.9 | (25.1; 41.7) |  |
| **Sickle cell status** |  |  |  |  |
| Normal | 267 | 31.4 | (24.4; 39.4) | 0.9095 |
| Sickle cell trait (HbAS) or disease (HbSS) | 46 | 32.2 | (20.0; 47.5) |  |
| **α-thalassemia** |  |  |  |  |
| Normal | 184 | 30.5 | (23.6; 38.5) | 0.5449 |
| α-thalassemia heterozygote/ homozygote | 101 | 35.4 | (22.8; 50.5) |  |
| **Vitamin A deficient** ^e^ |  |  |  |  |
| No (RBP≥0.7 μmol/L) | 270 | 27.3 | (20.8; 35.0) | **0.0003** |
| Yes (RBP<0.7 μmol/L) | 48 | 50.6 | (38.6; 62.6) |  |
| **Iron deficient** ^e^ |  |  |  |  |
| No (ferrtin≥12 μg/L) | 285 | 29.8 | (23.5; 37.1) | 0.1382 |
| Yes (ferritin<12 μg/L) | 33 | 41.5 | (25.2; 59.9) |  |
| **Stunting** |  |  |  |  |
| No (HAZ ≥-2SD) | 256 | 30.7 | (23.0; 39.7) | 0.6093 |
| Yes (HAZ <-2SD) | 57 | 34.2 | (21.3; 49.9) |  |
| **Wasting** |  |  |  |  |
| No (WHZ ≥-2SD) | 292 | 31.8 | (23.8; 41.0) | 0.6075 |
| Yes (WHZ <-2SD) | 18 | 26.3 | (11.6; 49.2) |  |
| **Underweight** |  |  |  |  |
| No (WHZ ≥-2SD) | 276 | 33.0 | (25.7; 41.1) | 0.8694 |
| Yes (WHZ <-2SD) | 42 | 31.5 | (16.0; 52.6) |  |
| **Inflammation categories** ^f^ |  |  |  |  |
| None | 151 | 17.6 | (12.7; 23.8) | **<0.0001** |
| Elevated CRP only | 11 | 41.6 | (17.0; 71.3) |  |
| Elevated CRP and AGP | 66 | 50.5 | (38.2; 62.8) |  |
| Elevated AGP only | 90 | 40.4 | (28.0; 54.2) |  |
| **Any inflammation** |  |  |  |  |
| None | 151 | 17.6 | (12.7; 23.8) | **<0.0001** |
| Any inflammation | 167 | 44.3 | (34.8; 54.3) |  |
| **Malaria status** ^g^ |  |  |  |  |
| Negative | 227 | 23.0 | (17.3; 29.9) | **<0.0001** |
| Positive | 96 | 62.2 | (45.5; 76.5) |  |
| **Diarrhea in past 2 weeks** |  |  |  |  |
| No | 253 | 29.2 | (22.5; 37.0) | **0.0622** |
| Yes | 70 | 43.8 | (28.0; 61.0) |  |
| **Cough in past 2 weeks** |  |  |  |  |
| No | 217 | 33.5 | (26.1; 41.9) | 0.6401 |
| Yes | 105 | 29.9 | (19.1; 43.5) |  |
| **Fever in past 2 weeks** |  |  |  |  |
| No | 192 | 22.0 | (15.9; 29.7) | **0.0013** |
| Yes | 131 | 48.2 | (35.9; 60.7) |  |
| **Lower respiratory infection in past 2 weeks** |  |  |  |  |
| No | 311 | 31.7 | (24.0; 40.6) | 0.3006 |
| Yes | 12 | 50.4 | (21.7; 78.8) |  |
| **Household sanitation** |  |  |  |  |
| Unimproved | 264 | 35.2 | (27.9; 43.3) | **0.0133** |
| Improved | 59 | 20.8 | (12.5; 32.6) |  |
| **Household owns soap** |  |  |  |  |
| No | 11 | 26.7 | (14.2; 44.5) | 0.5188 |
| Yes | 268 | 32.3 | (24.1; 41.6) |  |
| **Household has water at handwashing site** |  |  |  |  |
| No | 55 | 32.4 | (21.9; 45.0) | 0.9508 |
| Yes | 224 | 32.0 | (23.0; 42.5) |  |
| **Household wealth quintile** |  |  |  |  |
| Lowest | 72 | 51.3 | (37.0; 65.4) | **0.0013** |
| Second | 68 | 34.9 | (28.7; 41.6) |  |
| Middle | 65 | 38.5 | (27.7; 50.6) |  |
| Fourth | 61 | 28.7 | (16.9; 44.5) |  |
| Highest | 57 | 11.6 | (4.8; 25.3) |  |
| **Residence** |  |  |  |  |
| Urban | 152 | 26.5 | (17.1; 38.6) | **0.0723** |
| Rural | 171 | 39.9 | (31.2; 49.2) |  |
| Note: The n’s are un-weighted denominators for each subgroup; subgroups that do not sum to the total have missing data of missing data. | | | | |
| ^a^ Percentages weighted for unequal probability of selection. | | | | |
| ^b^ Anemia defined as hemoglobin < 110 g/L. | | | | |
| ^c^ CI=confidence interval, calculated taking into account the complex sampling design. | | | | |
| ^d^ Chi-square p-value <0.05 indicates that the proportion in at least one subgroup is statistically significantly different from the values in the other subgroups | | | | |
| ^e^ RBP [18] and ferritin [17] values adjusted for inflammation using Thurnham approach | | | | |
| ^f^ Elevated CRP and AGP defined as > 5mg/ L and >1 g/L, respectively | | | | |
| ^g^ Positive malaria status identified using rapid diagnostic tests during GMS data collection | | | | |

Supplementary Table 3. Bivariate associations between various risk factors and anemia in children in Ghana, Middle Stratum results

| ***Characteristic*** | ***Number of children in group*** | ***% Anemia ^a, b^*** | ***(95% CI) ^c^*** | ***P value ^d^*** |
| --- | --- | --- | --- | --- |
| **Age Group (in months)** |  |  |  |  |
| 6-11 | 51 | 39.2 | (26.9; 52.9) | 0.2628 |
| 12-23 | 118 | 29.1 | (21.2; 38.4) |  |
| 24-35 | 94 | 28.4 | (18.2; 41.5) |  |
| 36-47 | 98 | 28.3 | (16.8; 43.6) |  |
| 48-59 | 86 | 20.5 | (13.9; 29.2) |  |
| **Sex** |  |  |  |  |
| Male | 225 | 31.9 | (24.8; 40.0) | **0.0693** |
| Female | 220 | 24.6 | (17.5; 33.3) |  |
| **Sickle cell status** |  |  |  |  |
| Normal | 367 | 28.5 | (21.5; 36.7) | 0.5472 |
| Sickle cell trait (HbAS) or disease (HbSS) | 65 | 24.9 | (16.7; 35.4) |  |
| **α-thalassemia** |  |  |  |  |
| Normal | 295 | 24.5 | (18.2; 32.1) | **0.0869** |
| α-thalassemia heterozygote/ homozygote | 119 | 35.9 | (24.6; 49.0) |  |
| **Vitamin A deficient** ^e^ |  |  |  |  |
| No (RBP≥0.7 μmol/L) | 366 | 24.6 | (19.9; 30.1) | **0.0073** |
| Yes (RBP<0.7 μmol/L) | 75 | 41.4 | (27.0; 57.4) |  |
| **Iron deficient** ^e^ |  |  |  |  |
| No (ferrtin≥12 μg/L) | 357 | 24.0 | (18.0; 31.3) | **<0.0001** |
| Yes (ferritin<12 μg/L) | 84 | 44.8 | (34.2; 55.9) |  |
| **Stunting** |  |  |  |  |
| No (HAZ ≥-2SD) | 346 | 26.5 | (19.9; 34.2) | 0.1380 |
| Yes (HAZ <-2SD) | 96 | 34.0 | (25.4; 43.8) |  |
| **Wasting** |  |  |  |  |
| No (WHZ ≥-2SD) | 410 | 27.8 | (21.8; 34.8) | 0.8974 |
| Yes (WHZ <-2SD) | 28 | 29.3 | (12.1; 55.5) |  |
| **Underweight** |  |  |  |  |
| No (WHZ ≥-2SD) | 377 | 26.6 | (20.7; 33.5) | 0.1133 |
| Yes (WHZ <-2SD) | 62 | 34.6 | (24.3; 46.6) |  |
| **Inflammation categories** ^f^ |  |  |  |  |
| None | 237 | 15.8 | (10.2; 23.7) | **<0.0001** |
| Elevated CRP only | 13 | 38.4 | (13.9; 70.7) |  |
| Elevated CRP and AGP | 81 | 51.7 | (41.9; 61.2) |  |
| Elevated AGP only | 110 | 32.2 | (24.1; 41.5) |  |
| **Any inflammation** |  |  |  |  |
| None | 237 | 15.8 | (10.2; 23.7) | **0.0001** |
| Any inflammation | 204 | 40.4 | (33.5; 47.8) |  |
| **Malaria status** ^g^ |  |  |  |  |
| Negative | 299 | 23.2 | (16.2; 32.0) | **0.0005** |
| Positive | 88 | 44.1 | (38.6; 49.7) |  |
| **Diarrhea in past 2 weeks** |  |  |  |  |
| No | 337 | 29.5 | (22.5; 37.6) | 0.1537 |
| Yes | 110 | 24.3 | (18.1; 31.7) |  |
| **Cough in past 2 weeks** |  |  |  |  |
| No | 353 | 29.0 | (21.0; 38.4) | 0.6188 |
| Yes | 94 | 25.3 | (16.5; 36.6) |  |
| **Fever in past 2 weeks** |  |  |  |  |
| No | 311 | 26.0 | (19.5; 33.8) | **0.0373** |
| Yes | 136 | 33.3 | (25.6; 42.0) |  |
| **Lower respiratory infection in past 2 weeks** |  |  |  |  |
| No | 446 | 28.2 | (21.9; 35.5) | 0.5379 |
| Yes | 1 | 0.0 | -- |  |
| **Household sanitation** |  |  |  |  |
| Unimproved | 408 | 28.7 | (22.3; 36.1) | 0.3701 |
| Improved | 39 | 23.8 | (14.7; 36.1) |  |
| **Household owns soap** |  |  |  |  |
| No | 20 | 24.0 | (12.4; 41.5) | 0.4893 |
| Yes | 369 | 30.2 | (23.3; 38.1) |  |
| **Household has water at handwashing site** |  |  |  |  |
| No | 181 | 29.1 | (22.1; 37.3) | 0.7551 |
| Yes | 208 | 30.5 | (22.6; 39.8) |  |
| **Household wealth quintile** |  |  |  |  |
| Lowest | 96 | 34.3 | (22.7; 48.2) | **0.0468** |
| Second | 95 | 27.9 | (14.8; 46.4) |  |
| Middle | 107 | 36.3 | (28.1; 45.5) |  |
| Fourth | 74 | 21.8 | (13.6; 33.0) |  |
| Highest | 75 | 13.8 | (8.5; 21.5) |  |
| **Residence** |  |  |  |  |
| Urban | 193 | 21.1 | (14.1; 30.5) | **0.0418** |
| Rural | 254 | 33.9 | (25.8; 43.0) |  |
| Note: The n’s are un-weighted denominators for each subgroup; subgroups that do not sum to the total have missing data of missing data. | | | | |
| ^a^ Percentages weighted for unequal probability of selection. | | | | |
| ^b^ Anemia defined as hemoglobin < 110 g/L. | | | | |
| ^c^ CI=confidence interval, calculated taking into account the complex sampling design. | | | | |
| ^d^ Chi-square p-value <0.05 indicates that the proportion in at least one subgroup is statistically significantly different from the values in the other subgroups | | | | |
| ^e^ RBP [18] and ferritin [17] values adjusted for inflammation using Thurnham approach | | | | |
| ^f^ Elevated CRP and AGP defined as > 5mg/ L and >1 g/L, respectively | | | | |
| ^g^ Positive malaria status identified using rapid diagnostic tests during GMS data collection | | | | |

Supplementary Table 4. Bivariate associations between various risk factors and anemia in children in Ghana, North Stratum results

| ***Characteristic*** | ***Number of children in group*** | ***% Anemia ^a, b^*** | ***(95% CI) ^c^*** | ***P value ^d^*** |
| --- | --- | --- | --- | --- |
| **Age Group (in months)** |  |  |  |  |
| 6-11 | 34 | 74.1 | (53.7; 87.5) | **<0.0001** |
| 12-23 | 91 | 79.4 | (71.3; 85.6) |  |
| 24-35 | 94 | 58.8 | (45.8; 70.7) |  |
| 36-47 | 93 | 42.1 | (26.3; 59.7) |  |
| 48-59 | 90 | 25.7 | (16.7; 37.3) |  |
| **Sex** |  |  |  |  |
| Male | 191 | 59.6 | (50.7; 67.8) | **0.0359** |
| Female | 211 | 47.2 | (38.1; 56.5) |  |
| **Sickle cell status** |  |  |  |  |
| Normal | 356 | 52.6 | (45.6; 59.4) | 0.3985 |
| Sickle cell trait (HbAS) or disease (HbSS) | 31 | 63.6 | (36.5; 84.2) |  |
| **α-thalassemia** |  |  |  |  |
| Normal | 248 | 48.3 | (41.7; 54.9) | **0.0877** |
| α-thalassemia heterozygote/ homozygote | 130 | 59.2 | (46.3; 70.9) |  |
| **Vitamin A deficient** ^e^ |  |  |  |  |
| No (RBP≥0.7 μmol/L) | 283 | 51.7 | (43.9; 59.5) | 0.3456 |
| Yes (RBP<0.7 μmol/L) | 117 | 57.8 | (46.5; 68.3) |  |
| **Iron deficient** ^e^ |  |  |  |  |
| No (ferrtin≥12 μg/L) | 240 | 40.6 | (33.3; 48.4) | **<0.0001** |
| Yes (ferritin<12 μg/L) | 160 | 73.3 | (64.4; 80.6) |  |
| **Stunting** |  |  |  |  |
| No (HAZ ≥-2SD) | 296 | 48.1 | (40.0; 56.4) | **0.0046** |
| Yes (HAZ <-2SD) | 104 | 67.9 | (57.6; 76.7) |  |
| **Wasting** |  |  |  |  |
| No (WHZ ≥-2SD) | 357 | 52.7 | (45.1; 60.2) | 0.5882 |
| Yes (WHZ <-2SD) | 43 | 57.8 | (40.6; 73.3) |  |
| **Underweight** |  |  |  |  |
| No (WHZ ≥-2SD) | 319 | 50.4 | (42.5; 58.3) | 0.1676 |
| Yes (WHZ <-2SD) | 81 | 63.5 | (47.2; 77.1) |  |
| **Inflammation categories** ^f^ |  |  |  |  |
| None | 252 | 49.6 | (41.5; 57.7) | 0.2371 |
| Elevated CRP only | 15 | 65.0 | (32.1; 87.9) |  |
| Elevated CRP and AGP | 51 | 57.2 | (41.8; 71.4) |  |
| Elevated AGP only | 82 | 62.3 | (49.9; 73.2) |  |
| **Any inflammation** |  |  |  |  |
| None | 252 | 49.6 | (41.5; 57.7) | **0.0561** |
| Any inflammation | 148 | 60.8 | (51.0; 69.9) |  |
| **Malaria status** ^g^ |  |  |  |  |
| Negative | 353 | 51.2 | (44.3; 58.0) | **0.0846** |
| Positive | 49 | 69.7 | (47.0; 85.6) |  |
| **Diarrhea in past 2 weeks** |  |  |  |  |
| No | 292 | 48.0 | (40.6; 55.4) | **0.0343** |
| Yes | 109 | 68.0 | (53.5; 79.7) |  |
| **Cough in past 2 weeks** |  |  |  |  |
| No | 294 | 50.5 | (44.0; 56.8) | **0.0735** |
| Yes | 108 | 60.9 | (48.7; 71.8) |  |
| **Fever in past 2 weeks** |  |  |  |  |
| No | 270 | 49.6 | (42.9; 56.4) | **0.0255** |
| Yes | 132 | 61.3 | (50.8; 70.9) |  |
| **Lower respiratory infection in past 2 weeks** |  |  |  |  |
| No | 387 | 52.6 | (45.7; 59.5) | **0.0317** |
| Yes | 15 | 69.3 | (54.4; 81.0) |  |
| **Household sanitation** |  |  |  |  |
| Unimproved | 364 | 52.1 | (45.3; 58.8) | 0.2918 |
| Improved | 38 | 64.5 | (40.5; 82.9) |  |
| **Household owns soap** |  |  |  |  |
| No | 78 | 50.1 | (37.9; 62.4) | 0.3345 |
| Yes | 249 | 56.5 | (49.5; 63.2) |  |
| **Household has water at handwashing site** |  |  |  |  |
| No | 154 | 55.9 | (44.3; 66.9) | 0.8509 |
| Yes | 173 | 54.5 | (46.3; 62.5) |  |
| **Household wealth quintile** |  |  |  |  |
| Lowest | 252 | 50.7 | (41.8; 59.6) | 0.7763 |
| Second | 84 | 57.7 | (46.7; 68.1) |  |
| Middle | 41 | 55.4 | (27.2; 80.5) |  |
| Fourth | 19 | 64.2 | (36.1; 85.1) |  |
| Highest | 6 | 59.7 | (17.0; 91.5) |  |
| **Residence** |  |  |  |  |
| Urban | 87 | 49.8 | (37.5; 62.1) | 0.5506 |
| Rural | 315 | 54.2 | (46.2; 61.9) |  |
| Note: The n’s are un-weighted denominators for each subgroup; subgroups that do not sum to the total have missing data of missing data. | | | | |
| ^a^ Percentages weighted for unequal probability of selection. | | | | |
| ^b^ Anemia defined as hemoglobin < 110 g/L. | | | | |
| ^c^ CI=confidence interval, calculated taking into account the complex sampling design. | | | | |
| ^d^ Chi-square p-value <0.05 indicates that the proportion in at least one subgroup is statistically significantly different from the values in the other subgroups | | | | |
| ^e^ RBP [18] and ferritin [17] values adjusted for inflammation using Thurnham approach | | | | |
| ^f^ Elevated CRP and AGP defined as > 5mg/ L and >1 g/L, respectively | | | | |
| ^g^ Positive malaria status identified using rapid diagnostic tests during GMS data collection | | | | |

Supplementary Table 5. Bivariate associations between various risk factors and anemia in women in Ghana, National results

| **Characteristic** | ***Number of women in group*** | ***Anemia %^a, b^*** | ***(95% CI) ^c^*** | ***P value ^d^*** | |
| --- | --- | --- | --- | --- | --- |
| **Age group (in years)** |  |  |  |  | |
| 15-19 | 207 | 26.4 | (19.1; 35.2) | 0.5942 | |
| 20-24 | 169 | 20.9 | (14.2; 29.6) |  | |
| 25-29 | 178 | 23.1 | (17.0; 30.5) |  | |
| 30-34 | 169 | 23.0 | (16.4; 31.3) |  | |
| 35-39 | 113 | 15.5 | (7.8; 28.5) |  | |
| 40-44 | 103 | 16.9 | (10.0; 27.1) |  | |
| 45-49 | 46 | 20.0 | (9.3; 37.9) |  | |
| **Minimum dietary diversity** |  |  |  |  | |
| No | 509 | 23.9 | (19.4; 29.0) | 0.1184 | |
| Yes | 490 | 19.3 | (15.8; 23.2) |  | |
| **Sickle cell** |  |  |  |  | |
| Normal (AA) | 413 | 21.2 | (16.9; 26.2) | 0.2208 | |
| Sickle cell trait (HbAS) or disease (HbSS | 59 | 28.4 | (18.3; 41.2) |  | |
| **α-thalassemia** |  |  |  |  | |
| Normal | 307 | 21.8 | (17.0; 27.5) | 0.8914 | |
| α-thalassemia heterozygous/ homozygous | 160 | 22.5 | (15.6; 31.4) |  | |
| **Inflammation** ^e^ |  |  |  |  | |
| None | 798 | 19.3 | (15.9; 23.2) | **0.0002** | |
| Elevated CRP only | 63 | 8.3 | (3.3; 19.4) |  | |
| Elevated CRP and AGP | 56 | 40.1 | (27.8; 53.7) |  | |
| Elevated AGP only | 59 | 33.0 | (21.3; 47.2) |  | |
| **Inflammation** ^e^ |  |  |  |  | |
| None | 798 | 19.3 | (15.9; 23.2) | **0.0182** | |
| Elevated CRP and/or AGP | 178 | 27.3 | (21.7; 33.7) |  | |
| **Malaria status** ^f^ |  |  |  |  | |
| Negative | 869 | 21.4 | (17.8; 25.5) | 0.9354 | |
| Positive | 78 | 21.9 | (12.9; 34.7) |  | |
| **Folate status** ^g^ |  |  |  |  | |
| Deficient (<10 ηmol/L) | 257 | 23.5 | (18.2; 29.9) | 0.7409 | |
| Sufficient (≥10ηmol/L) | 210 | 22.1 | (16.6; 28.9) |  | |
| **B_12_ status** ^g^ |  |  |  |  | |
| Deficient (<148pmol/L) | 38 | 6.1 | (1.8; 18.6) | **0.0310** | |
| Marginal (148-220pmol/L) | 55 | 17.8 | (9.4; 31.1) |  | |
| Sufficient (>220pmol/L) | 372 | 24.8 | (20.3; 30.1) |  | |
| **Vitamin A insufficiency** ^h^ |  |  |  |  | |
| No (RBP≥1.05 μmol/L) | 857 | 17.6 | (15.1; 20.4) | **<0.0001** | |
| Yes (RBP<1.05 μmol/L) | 119 | 45.3 | (36.3; 54.6) |  | |
| **Iron deficient** ^h^ |  |  |  |  | |
| No (ferrtin≥15 μg/L) | 845 | 13.9 | (11.4; 17.0) | **<0.0001** | |
| Yes (ferritin<15 μg/L) | 131 | 64.8 | (55.1; 73.4) |  | |
| **Underweight** |  |  |  |  | |
| No (BMI≥ 18.5) | 906 | 20.8 | (17.8; 24.0) | 0.1488 | |
| Yes (BMI< 18.5) | 83 | 28.7 | (18.0; 42.6) |  | |
| **Overweight** |  |  |  |  | |
| No (BMI<25.9) | 635 | 24.1 | (19.8; 29.0) | **0.0661** | |
| Yes (BMI 25.0-29.9) | 228 | 17.6 | (12.8; 23.8) |  | |
| **Obesity** |  |  |  |  | |
| No (BMI<29.9) | 863 | 22.2 | (18.5; 26.4) | 0.2958 | |
| Yes (BMI≥30.0) | 126 | 16.4 | (9.7; 26.5) |  | |
| **Overweight or Obesity** |  |  |  |  | |
| No (BMI< 25.0) | 635 | 24.1 | (19.8; 29.0) | **0.0438** | |
| Yes (BMI≥25.0) | 354 | 17.2 | (13.2, 22.1) |  | |
| **Household sanitation** |  |  |  |  | |
| Unimproved | 889 | 20.9 | (18.1; 24.1) | 0.1402 | |
| Improved | 110 | 26.8 | (19.1; 36.2) |  | |
| **Household owns soap** |  |  |  |  | |
| No | 87 | 22.9 | (12.8; 37.6) | 0.7019 | |
| Yes | 769 | 20.4 | (17.2; 24.0) |  | |
| **Household has water at handwashing site** |  |  |  |  | |
| No | 341 | 21.9 | (17.2; 27.5) | 0.4733 | |
| Yes | 515 | 19.8 | (16.5; 23.6) |  | |
| **Household wealth quintile** |  |  |  |  | |
| Lowest | 278 | 21.2 | (14.7; 29.5) | 0.4693 | |
| Second | 211 | 22.4 | (16.8; 29.2) |  | |
| Middle | 187 | 25.8 | (18.6; 34.6) |  | |
| Fourth | 161 | 21.4 | (15.8; 28.4) |  | |
| Highest | 162 | 17.0 | (12.1; 23.3) |  | |
| **Stratum** |  |  |  |  | |
| Southern Belt | 303 | 23.9 | (18.9; 29.8) | **0.0301** | |
| Middle Belt | 404 | 17.5 | (14.1; 21.5) |  | |
| Northern Belt | 292 | 27.6 | (20.4; 36.2) |  | |
| **Residence** |  |  |  |  | |
| Urban | 436 | 21.6 | (17.5; 26.4) | 0.9642 | |
| Rural | 563 | 21.8 | (17.4; 26.8) |  | |
| Note: The n’s are un-weighted denominators for each subgroup; subgroups that do not sum to the total have missing data of missing data. | | | | |  |
| ^a^ Percentages weighted for unequal probability of selection. | | | | |  |
| ^b^ Anemia defined as hemoglobin < 120 g/L adjusted for smoking. | | | | |  |
| ^c^ CI=confidence interval, calculated taking into account the complex sampling design. | | | | |  |
| ^d^ Chi-square p-value <0.05 indicates that the proportion in at least one subgroup is statistically significantly different from the values in the other subgroups. | | | | |  |
| ^e^ Inflammation defined as elevated CRP (>5mg/L) and/ elevated AGP (>1g/L) | | | | |  |
| ^f^ Malaria status identified using rapid diagnostic tests during GMS data collection | | | | |  |
| ^g^ Measured in a sub- sample  ^h^ RBP [18] and ferritin [17] values adjusted according to Thurnham | | | | |  |

Supplementary Table 6. Bivariate associations between various risk factors and anemia in women in Ghana, South Stratum results

| **Characteristic** | ***Number of women in group*** | ***Anemia %^a, b^*** | ***(95% CI) ^c^*** | ***P value ^d^*** | |
| --- | --- | --- | --- | --- | --- |
| **Age group (in years)** |  |  |  |  | |
| 15-19 | 62 | 25.1 | (15.0; 39.0) | 0.3249 | |
| 20-24 | 45 | 31.9 | (16.6; 52.6) |  | |
| 25-29 | 61 | 31.0 | (19.5; 45.5) |  | |
| 30-34 | 56 | 15.7 | (7.7; 29.5) |  | |
| 35-39 | 35 | 10.9 | (4.3; 24.8) |  | |
| 40-44 | 31 | 25.6 | (12.0; 46.4) |  | |
| 45-49 | 12 | 24.6 | (5.6; 64.3) |  | |
| **Minimum dietary diversity** |  |  |  |  | |
| No | 184 | 27.1 | (19.4; 36.5) | 0.1228 | |
| Yes | 118 | 18.8 | (13.6; 25.6) |  | |
| **Sickle cell** |  |  |  |  | |
| Normal (AA) | 122 | 22.1 | (15.6; 30.4) | 0.2004 | |
| Sickle cell trait (HbAS) or disease (HbSS | 17 | 34.9 | (17.3; 57.8) |  | |
| **α-thalassemia** |  |  |  |  | |
| Normal | 80 | 26.7 | (19.0; 36.1) | 0.4892 | |
| α-thalassemia heterozygous/ homozygous | 57 | 20.1 | (9.1; 38.7) |  | |
| **Inflammation** ^e^ |  |  |  |  | |
| None | 235 | 21.8 | (17.2; 27.3) | **0.0203** | |
| Elevated CRP only | 20 | 9.0 | (1.8; 34.9) |  | |
| Elevated CRP and AGP | 23 | 40.0 | (25.6; 56.4) |  | |
| Elevated AGP only | 20 | 38.0 | (20.7; 59.2) |  | |
| **Inflammation** ^e^ |  |  |  |  | |
| None | 235 | 21.8 | (17.2; 27.3) | **0.0347** | |
| Elevated CRP and/or AGP | 63 | 30.4 | (21.3; 41.2) |  | |
| **Malaria status** ^f^ |  |  |  |  | |
| Negative | 282 | 24.2 | (18.7; 30.7) | 0.7848 | |
| Positive | 20 | 20.5 | (5.3; 54.1) |  | |
| **Folate status** ^g^ |  |  |  |  | |
| Sufficient (≥10ηmol/L) | 69 | 25.9 | (16.4; 38.4) | 0.6461 | |
| Deficient (<10 ηmol/L) | 70 | 23.0 | (15.0; 33.7) |  | |
| **B_12_ status** ^g^ |  |  |  |  | |
| Deficient (<148pmol/L) | 5 | 17.2 | (1.8; 70.1) | 0.9334 | |
| Marginal (148-220pmol/L) | 9 | 24.5 | (6.9; 58.9) |  | |
| Sufficient (>220pmol/L) | 124 | 24.8 | (16.8; 34.9) |  | |
| **Vitamin A insufficiency** ^h^ |  |  |  |  | |
| No (RBP≥1.05 μmol/L) | 272 | 21.4 | (17.1; 26.5) | **0.0014** | |
| Yes (RBP<1.05 μmol/L) | 26 | 48.1 | (27.5; 69.3) |  | |
| **Iron deficient** ^h^ |  |  |  |  | |
| No (ferrtin≥15 μg/L) | 260 | 16.8 | (12.2; 22.9) | **<0.0001** | |
| Yes (ferritin<15 μg/L) | 38 | 67.0 | (50.9; 80.0) |  | |
| **Underweight** |  |  |  |  | |
| No (BMI≥ 18.5) | 281 | 23.6 | (18.7; 29.4) | 0.4855 | |
| Yes (BMI< 18.5) | 20 | 31.5 | (11.9; 61.2) |  | |
| **Overweight** |  |  |  |  | |
| No (BMI<25.9) | 167 | 29.9 | (23.5; 37.2) | 0.1612 | |
| Yes (BMI 25.0-29.9) | 83 | 21.2 | (12.1; 34.6) |  | |
| **Obesity** |  |  |  |  | |
| No (BMI<29.9) | 250 | 26.9 | (20.7; 34.1) | **0.0184** | |
| Yes (BMI≥30.0) | 51 | 12.4 | (6.0; 23.9) |  | |
| **Overweight or Obesity** |  |  |  |  | |
| No (BMI< 25.0) | 167 | 29.9 | (23.5; 37.2) | **0.0118** | |
| Yes (BMI≥25.0) | 134 | 17.6 | (11.1; 26.9) |  | |
| **Household sanitation** |  |  |  |  | |
| Unimproved | 247 | 23.5 | (18.5; 29.4) | 0.6777 | |
| Improved | 55 | 25.6 | (16.4; 37.7) |  | |
| **Household owns soap** |  |  |  |  | |
| No | 14 | 24.7 | (7.1; 58.5) | 0.8451 | |
| Yes | 237 | 22.0 | (17.2; 27.7) |  | |
| **Household has water at handwashing site** |  |  |  |  | |
| No | 63 | 21.0 | (14.1; 30.1) | 0.7019 | |
| Yes | 188 | 22.7 | (17.7; 28.6) |  | |
| **Household wealth quintile** |  |  |  |  | |
| Lowest | 49 | 24.8 | (17.8; 33.4) | 0.1306 | |
| Second | 60 | 25.9 | (14.1; 42.8) |  | |
| Middle | 55 | 35.0 | (24.9; 46.6) |  | |
| Fourth | 63 | 19.4 | (11.6; 30.6) |  | |
| Highest | 75 | 18.1 | (10.9; 28.6) |  | |
| **Residence** |  |  |  |  | |
| Urban | 171 | 24.8 | (17.9; 33.3) |  | |
| Rural | 131 | 22.5 | (16.3; 30.1) |  | |
| Note: The n’s are un-weighted denominators for each subgroup; subgroups that do not sum to the total have missing data of missing data. | | | | |  |
| ^a^ Percentages weighted for unequal probability of selection. | | | | |  |
| ^b^ Anemia defined as hemoglobin < 120 g/L adjusted for smoking. | | | | |  |
| ^c^ CI=confidence interval, calculated taking into account the complex sampling design. | | | | |  |
| ^d^ Chi-square p-value <0.05 indicates that the proportion in at least one subgroup is statistically significantly different from the values in the other subgroups. | | | | |  |
| ^e^ Inflammation defined as elevated CRP (>5mg/L) and/ elevated AGP (>1g/L) | | | | |  |
| ^f^ Malaria status identified using rapid diagnostic tests during GMS data collection | | | | |  |
| ^g^ Measured in a sub- sample  ^h^ RBP [18] and ferritin [17] values adjusted according to Thurnham | | | | |  |

Supplementary Table 7. Bivariate associations between various risk factors and anemia in women in Ghana, Middle Stratum results

| **Characteristic** | ***Number of women in group*** | ***Anemia %^a, b^*** | ***(95% CI) ^c^*** | ***P value ^d^*** | |
| --- | --- | --- | --- | --- | --- |
| **Age group (in years)** |  |  |  |  | |
| 15-19 | 88 | 20.2 | (11.4; 33.2) | 0.3394 | |
| 20-24 | 73 | 11.1 | (6.1; 19.3) |  | |
| 25-29 | 70 | 17.5 | (9.8; 29.5) |  | |
| 30-34 | 58 | 28.1 | (16.7; 43.2) |  | |
| 35-39 | 51 | 14.3 | (5.7; 31.3) |  | |
| 40-44 | 46 | 11.6 | (5.0; 24.8) |  | |
| 45-49 | 18 | 21.0 | (6.2; 52.0) |  | |
| **Minimum dietary diversity** |  |  |  |  | |
| No | 168 | 16.8 | (11.1; 24.6) | 0.7985 | |
| Yes | 237 | 17.9 | (13.2; 24.0) |  | |
| **Sickle cell** |  |  |  |  | |
| Normal (AA) | 160 | 18.3 | (11.7; 27.6) | 0.6300 | |
| Sickle cell trait (HbAS) or disease (HbSS | 30 | 22.1 | (10.5; 40.7) |  | |
| **α-thalassemia** |  |  |  |  | |
| Normal | 130 | 17.8 | (11.0; 27.4) | 0.6272 | |
| α-thalassemia heterozygous/ homozygous | 58 | 21.1 | (12.9; 32.5) |  | |
| **Inflammation** ^e^ |  |  |  |  | |
| None | 310 | 12.7 | (9.1; 17.5) | **0.0131** | |
| Elevated CRP only | 30 | 9.9 | (2.9; 28.5) |  | |
| Elevated CRP and AGP | 26 | 37.4 | (16.8; 63.8) |  | |
| Elevated AGP only |  |  |  |  | |
| **Inflammation** ^e^ |  |  |  |  | |
| None | 310 | 12.7 | (9.1; 17.5) | **0.0039** | |
| Elevated CRP and/or AGP | 85 | 26.3 | (18.9; 35.4) |  | |
| **Malaria status** ^f^ |  |  |  |  | |
| Negative | 312 | 15.4 | (10.9; 21.5) | 0.4062 | |
| Positive | 41 | 22.1 | (11.3; 38.7) |  | |
| **Folate status** ^g^ |  |  |  |  | |
| Sufficient (≥10ηmol/L) | 82 | 19.1 | (10.8; 31.5) | 0.8073 | |
| Deficient (<10 ηmol/L) | 106 | 20.8 | (12.9; 31.9) |  | |
| **B_12_ status** ^g^ |  |  |  |  | |
| Deficient (<148pmol/L) | 12 | 0.0 | -- | **0.0462** | |
| Marginal (148-220pmol/L) | 24 | 6.9 | (1.3; 29.3) |  | |
| Sufficient (>220pmol/L) | 152 | 24.0 | (17.6; 31.9) |  | |
| **Vitamin A insufficiency** ^h^ |  |  |  |  | |
| No (RBP≥1.05 μmol/L) | 360 | 13.7 | (10.9; 17.1) | **0.0006** | |
| Yes (RBP<1.05 μmol/L) | 35 | 38.5 | (23.5; 56.0) |  | |
| **Iron deficient** ^h^ |  |  |  |  | |
| No (ferrtin≥15 μg/L) | 353 | 11.2 | (8.3; 15.1) | **<0.0001** | |
| Yes (ferritin<15 μg/L) | 42 | 55.6 | (43.1; 67.3) |  | |
| **Underweight** |  |  |  |  | |
| No (BMI≥ 18.5) | 371 | 16.1 | (13.0; 19.9) | 0.2540 | |
| Yes (BMI< 18.5) | 30 | 26.1 | (10.5; 51.4) |  | |
| **Overweight** |  |  |  |  | |
| No (BMI<25.9) | 240 | 16.9 | (11.3; 24.5) | 0.6238 | |
| Yes (BMI 25.0-29.9) | 100 | 14.7 | (9.6; 21.9) |  | |
| **Obesity** |  |  |  |  | |
| No (BMI<29.9) | 340 | 16.2 | (11.9; 21.8) | 0.5720 | |
| Yes (BMI≥30.0) | 61 | 20.8 | (9.8; 38.9) |  | |
| **Overweight or Obesity** |  |  |  |  | |
| No (BMI< 25.0) | 240 | 16.9 | (11.3; 24.5) | 0.9970 | |
| Yes (BMI≥25.0) | 161 | 16.9 | (11.6; 23.9) |  | |
| **Household sanitation** |  |  |  |  | |
| Unimproved | 369 | 16.9 | (13.7; 20.8) | 0.3817 | |
| Improved | 36 | 22.8 | (10.9; 41.4) |  | |
| **Household owns soap** |  |  |  |  | |
| No | 22 | 22.1 | (8.3; 46.8) | 0.6056 | |
| Yes | 343 | 16.9 | (12.7; 22.1) |  | |
| **Household has water at handwashing site** |  |  |  |  | |
| No | 159 | 19.4 | (13.7; 26.7) | 0.3191 | |
| Yes | 206 | 15.7 | (11.7; 20.8) |  | |
| **Household wealth quintile** |  |  |  |  | |
| Lowest | 56 | 8.6 | (3.3; 20.6) | 0.5130 | |
| Second | 91 | 17.5 | (10.7; 27.4) |  | |
| Middle | 98 | 18.6 | (11.0; 29.7) |  | |
| Fourth | 79 | 21.9 | (13.8; 33.0) |  | |
| Highest | 81 | 15.8 | (9.8; 24.4) |  | |
| **Residence** |  |  |  |  | |
| Urban | 189 | 16.0 | (12.1; 20.8) | 0.4492 | |
| Rural | 216 | 18.7 | (13.4; 25.5) |  | |
| Note: The n’s are un-weighted denominators for each subgroup; subgroups that do not sum to the total have missing data of missing data. | | | | |  |
| ^a^ Percentages weighted for unequal probability of selection. | | | | |  |
| ^b^ Anemia defined as hemoglobin < 120 g/L adjusted for smoking. | | | | |  |
| ^c^ CI=confidence interval, calculated taking into account the complex sampling design. | | | | |  |
| ^d^ Chi-square p-value <0.05 indicates that the proportion in at least one subgroup is statistically significantly different from the values in the other subgroups. | | | | |  |
| ^e^ Inflammation defined as elevated CRP (>5mg/L) and/ elevated AGP (>1g/L) | | | | |  |
| ^f^ Malaria status identified using rapid diagnostic tests during GMS data collection | | | | |  |
| ^g^ Measured in a sub- sample  ^h^ RBP [18] and ferritin [17] values adjusted according to Thurnham | | | | |  |

Supplementary Table 8. Bivariate associations between various risk factors and anemia in women in Ghana, North Stratum results

| **Characteristic** | ***Number of women in group*** | ***Anemia %^a, b^*** | ***(95% CI) ^c^*** | ***P value ^d^*** | |
| --- | --- | --- | --- | --- | --- |
| **Age group (in years)** |  |  |  |  | |
| 15-19 | 64 | 40.3 | (24.9; 57.9) | 0.4588 | |
| 20-24 | 52 | 26.7 | (13.6; 45.9) |  | |
| 25-29 | 49 | 22.1 | (9.0; 44.7) |  | |
| 30-34 | 56 | 27.4 | (15.9; 43.1) |  | |
| 35-39 | 27 | 27.9 | (5.1; 73.5) |  | |
| 40-44 | 26 | 12.9 | (4.3; 32.5) |  | |
| 45-49 | 16 | 12.8 | (3.9; 34.8) |  | |
| **Minimum dietary diversity** |  |  |  |  | |
| No | 157 | 29.9 | (22.4; 38.7) | 0.1668 | |
| Yes | 135 | 24.3 | (16.1; 35.0) |  | |
| **Sickle cell** |  |  |  |  | |
| Normal (AA) | 131 | 25.5 | (18.3; 34.3) | 0.3857 | |
| Sickle cell trait (HbAS) or disease (HbSS | 12 | 37.3 | (15.6; 65.7) |  | |
| **α-thalassemia** |  |  |  |  | |
| Normal | 97 | 23.7 | (13.6; 38.0) | 0.5799 | |
| α-thalassemia heterozygous/ homozygous | 45 | 31.3 | (15.0; 54.0) |  | |
| **Inflammation** ^e^ |  |  |  |  | |
| None | 253 | 28.7 | (19.9; 39.6) | **0.0913** | |
| Elevated CRP only | 13 | 0.0 | -- |  | |
| Elevated CRP and AGP | 7 | 58.8 | (21.0; 88.4) |  | |
| Elevated AGP only | 10 | 21.1 | (6.0; 52.8) |  | |
| **Inflammation** ^e^ |  |  |  |  | |
| None | 253 | 28.7 | (19.9; 39.6) | 0.4543 | |
| Elevated CRP and/or AGP | 30 | 20.3 | (8.5; 41.1) |  | |
| **Malaria status** ^f^ |  |  |  |  | |
| Negative | 275 | 27.8 | (19.9; 37.5) | 0.8190 | |
| Positive | 17 | 24.2 | (6.3; 60.1) |  | |
| **Folate status** ^g^ |  |  |  |  | |
| Sufficient (≥10ηmol/L) | 59 | 22.1 | (14.5; 32.2) | 0.2366 | |
| Deficient (<10 ηmol/L) | 81 | 31.0 | (19.6; 45.2) |  | |
| **B_12_ status** ^g^ |  |  |  |  | |
| Deficient (<148pmol/L) | 21 | 7.7 | (1.8; 27.5) | 0.1497 | |
| Marginal (148-220pmol/L) | 22 | 34.1 | (16.3; 57.9) |  | |
| Sufficient (>220pmol/L) | 96 | 27.1 | (18.3; 38.2) |  | |
| **Vitamin A insufficiency** ^h^ |  |  |  |  | |
| No (RBP≥1.05 μmol/L) | 225 | 20.3 | (13.8; 28.9) | **0.0003** | |
| Yes (RBP<1.05 μmol/L) | 58 | 49.2 | (37.0; 61.4) |  | |
| **Iron deficient** ^h^ |  |  |  |  | |
| No (ferrtin≥15 μg/L) | 232 | 15.3 | (9.0; 25.0) | **<0.0001** | |
| Yes (ferritin<15 μg/L) | 51 | 73.0 | (50.7; 87.6) |  | |
| **Underweight** |  |  |  |  | |
| No (BMI≥ 18.5) | 254 | 26.7 | (18.6; 36.7) | 0.6919 | |
| Yes (BMI< 18.5) | 33 | 30.0 | (18.1; 45.3) |  | |
| **Overweight** |  |  |  |  | |
| No (BMI<25.9) | 228 | 29.4 | (21.2; 39.2) | **0.0625** | |
| Yes (BMI 25.0-29.9) | 45 | 17.1 | (9.8; 28.3) |  | |
| **Obesity** |  |  |  |  | |
| No (BMI<29.9) | 273 | 27.6 | (20.2; 36.5) | 0.3358 | |
| Yes (BMI≥30.0) | 14 | 14.0 | (2.5; 50.9) |  | |
| **Overweight or Obesity** |  |  |  |  | |
| No (BMI< 25.0) | 228 | 29.4 | (21.2; 39.2) | **0.0455** | |
| Yes (BMI≥25.0) | 59 | 16.5 | (9.2; 27.7) |  | |
| **Household sanitation** |  |  |  |  | |
| Unimproved | 273 | 26.3 | (18.9; 35.2) | 0.1059 | |
| Improved | 19 | 46.0 | (22.9; 71.0) |  | |
| **Household owns soap** |  |  |  |  | |
| No | 51 | 22.9 | (8.2; 50.0) | 0.6547 | |
| Yes | 189 | 28.0 | (19.9; 37.9) |  | |
| **Household has water at handwashing site** |  |  |  |  | |
| No | 119 | 29.0 | (16.3; 46.1) | 0.6882 | |
| Yes | 121 | 25.0 | (14.6; 39.2) |  | |
| **Household wealth quintile** |  |  |  |  | |
| Lowest | 173 | 24.7 | (15.3; 37.3) | 0.3126 | |
| Second | 60 | 30.6 | (20.9; 42.3) |  | |
| Middle | 35 | 39.1 | (24.9; 55.6) |  | |
| Fourth | 19 | 31.1 | (17.3; 49.3) |  | |
| Highest | 5 | 17.5 | (4.6; 48.5) |  | |
| **Residence** |  |  |  |  | |
| Urban | 76 | 31.4 | (22.3; 42.3) | 0.4892 | |
| Rural | 216 | 26.4 | (17.2; 38.2) |  | |
| Note: The n’s are un-weighted denominators for each subgroup; subgroups that do not sum to the total have missing data of missing data. | | | | |  |
| ^a^ Percentages weighted for unequal probability of selection. | | | | |  |
| ^b^ Anemia defined as hemoglobin < 120 g/L adjusted for smoking. | | | | |  |
| ^c^ CI=confidence interval, calculated taking into account the complex sampling design. | | | | |  |
| ^d^ Chi-square p-value <0.05 indicates that the proportion in at least one subgroup is statistically significantly different from the values in the other subgroups. | | | | |  |
| ^e^ Inflammation defined as elevated CRP (>5mg/L) and/ elevated AGP (>1g/L) | | | | |  |
| ^f^ Malaria status identified using rapid diagnostic tests during GMS data collection | | | | |  |
| ^g^ Measured in a sub- sample  ^h^ RBP [18] and ferritin [17] values adjusted according to Thurnham | | | | |  |
